# Saffron Pre-Treatment Promotes Reduction in Tissue Inflammatory Profiles and Alters Microbiome Composition in Experimental Colitis Mice

**DOI:** 10.3390/molecules26113351

**Published:** 2021-06-02

**Authors:** Suhrid Banskota, Hassan Brim, Yun Han Kwon, Gulshan Singh, Sidhartha R. Sinha, Huaqing Wang, Waliul I. Khan, Hassan Ashktorab

**Affiliations:** 1 Farncombe Family Digestive Health Research Institute, McMaster University, 1280 Main St. W, Hamilton, ON L8S 4K1, Canada; banskots@mcmaster.ca (S.B.); yyoon90@gmail.com (Y.H.K.); wanghu@mcmaster.ca (H.W.); 2 Department of Pathology and Molecular Medicine, McMaster University, Hamilton, ON L8S 4K1, Canada; 3 Department of Pathology, Cancer Center, Howard University College of Medicine, Washington, DC 20059, USA; hbrim@howard.edu; 4 Division of Gastroenterology and Hepatology, Stanford University, Stanford, CA 94305, USA; gsingh10@stanford.edu (G.S.); sidsinha@stanford.edu (S.R.S.); 5 Department of Medicine, Gastroenterology Division, Cancer Center, Howard University College of Medicine, Washington, DC 20059, USA

**Keywords:** inflammatory bowel disease, saffron, gut microbiota, colitis, cytokines

## Abstract

Inflammatory bowel disease (IBD) is a chronic inflammatory condition of the gastrointestinal tract with an incompletely understood pathogenesis. Long-standing colitis is associated with increased risk of colon cancer. Despite the availability of various anti-inflammatory and immunomodulatory drugs, many patients fail to respond to pharmacologic therapy and some experience drug-induced adverse events. Dietary supplements, particularly saffron (*Crocus sativus*), have recently gained an appreciable attention in alleviating some symptoms of digestive diseases. In our study, we investigated whether saffron may have a prophylactic effect in a murine colitis model. Saffron pre-treatment improved the gross and histopathological characteristics of the colonic mucosa in murine experimental colitis. Treatment with saffron showed a significant amelioration of colitis when compared to the vehicle-treated mice group. Saffron treatment significantly decreased secretion of serotonin and pro-inflammatory cytokines, such as TNF-α, IL-1β, and IL-6, in the colon tissues by suppressing the nuclear translocation of NF-κB. The gut microbiome analysis revealed distinct clusters in the saffron-treated and untreated mice in dextran sulfate sodium (DSS)-induced colitis by visualization of the Bray–Curtis diversity by principal coordinates analysis (PCoA). Furthermore, we observed that, at the operational taxonomic unit (OTU) level, Cyanobacteria were depleted, while short-chain fatty acids (SCFAs), such as isobutyric acid, acetic acid, and propionic acid, were increased in saffron-treated mice. Our data suggest that pre-treatment with saffron inhibits DSS-induced pro-inflammatory cytokine secretion, modulates gut microbiota composition, prevents the depletion of SCFAs, and reduces the susceptibility to colitis.

## 1. Introduction

Inflammatory bowel disease (IBD) is a chronic relapsing immune-inflammatory condition of the gastrointestinal (GI) tract with increasing prevalence worldwide [1]. IBD is broadly classified into Crohn’s disease (CD) and ulcerative colitis (UC) on the basis of their clinical presentation, but the risk factors implicated on the pathogenesis of both CD and UC are similar [2]. The etiology of IBD is complex and various studies suggest that its pathogenesis is associated with a dysregulated immune response, genetic factors, gut microbiota, and environmental factors [2]. Various immunosuppressive synthetic drugs and biologics, such as salicylates, corticosteroids, tumor necrosis factor (TNF) blockers, and vedolizumab, are available as therapies for IBD, and many additional options are in the pipeline [3,4,5]. Clinical data suggest that these therapies are limited in managing the disease in some patients, while some fail to respond over time. Moreover, failure in managing IBD over the long run deteriorates the inflammatory conditions and increases the risk of developing colon cancer [6,7,8,9,10]. Therefore, alternative approaches in preventing the induction or perpetuation of intestinal inflammation are important in decreasing the incidence of IBD.

Natural products, such as berberine, baicalein, curcumin, bromelain, and their chemical constituents, are reported to be effective in treating IBD, and the mechanism of action involved in ameliorating inflammation has been widely studied [11]. Saffron (*Crocus sativus*) has been used as a spice and for health management since ancient times and is reported to play a key role in treatment of different digestive system disorders [12,13,14,15]. Saffron, by virtue of its potent antioxidant property, showed a significant decrease in lipoprotein oxidation susceptibility (LOS) in human subjects and was evaluated as a promising anti-obesity drug [16]. Crocin, a biologically active carotenoid constituent of saffron, was demonstrated to protect against DSS-induced colitis in C57BL/6 J mice and suppressed tumor growth in ApcMinC/Gpt mice by suppressing NF-κB-mediated inflammation [17]. NF-κB, an oxidative stress sensitive transcription factor, is associated with tissue induction of pro-inflammatory cytokines, such as tumor necrosis factor (TNF)-α, interleukin (IL)-6, and IL-1β [18,19].

Alteration in the enterochromaffin (EC) cell numbers and intestinal 5-HT content has been observed in experimental colitis and in both UC and CD patients [20,21]. The altered 5-HT plays a key role in the activation and transportation of immune cells to produce proinflammatory cytokines by increasing angiogenesis [22,23,24]. Previous studies, including ours, demonstrated that excessive serotonin (5-HT) secreted from EC cells during DSS-induced inflammation in the gut plays an important role in the modulation of gut microbial composition as well as gut function [25,26]. The human GI tract is colonized with 1 × 10^14^ colony-forming units of bacteria and the colonization occurs soon after birth [27]. Accumulating evidence suggest that the gut microbiota has an important role on the pathogenesis of IBD. We previously demonstrated that 5-HT regulates the growth of bacteria in a species-dependent manner and selects for a more colitogenic microbiota [26]. The effect of saffron on the altered 5-HT and composition of gut microbiota in DSS-treated mice has not been evaluated so far.

The current study was conducted to investigate whether saffron has prophylactic effects on an experimental colitis mice model by evaluating the secretion of pro-inflammatory cytokines such as TNF-α, IL-6, and IL-1β in colon tissue, the colonic 5-HT level, assessing the cecal microbiota composition, and analyzing the changes in short-chain fatty acids in feces. The findings from this study will shed light on the translational perspective of the protective effect of saffron in human IBD.

## 2. Results

### 2.1. Saffron Alleviated DSS-Induced Colitis in Mice

**Figure 1:**
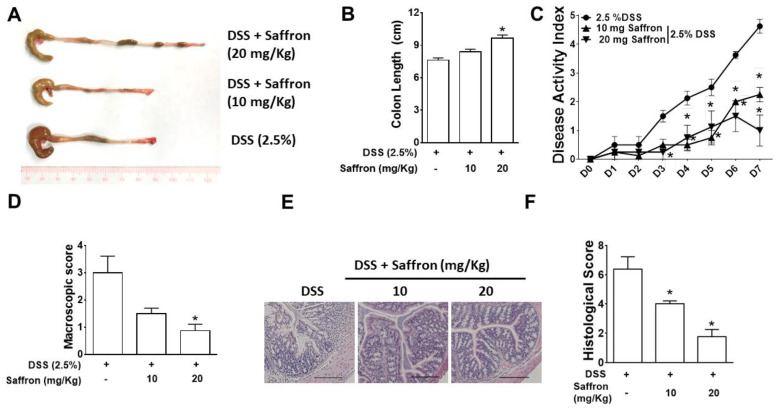
Saffron reduced the severity of DSS-induced colitis in mice. Mice were orally gavaged with saffron (10 mg/Kg and 20 mg/Kg) or the vehicle for 4 days before the administration of 2.5% DSS in their drinking water and continued for 7 days along with the DSS. Inflammation was assessed by (**A**) macroscopic appearance of the colon tissue; (**B**) colon length; (**C**) the disease activity index; (**D**) the macroscopic score; (**E**) representative micrographs of H&E-stained colon cross-sections on day 7 post-DSS, bar = 100 µM; and (**F**) the histological score. Data represent the mean ± SEM (*n* = 4/group). * *p* < 0.05, compared to the vehicle-treated mice.

To investigate the prophylactic effect of saffron in mice, C57BL/6 mice were orally gavaged with saffron (10 mg/kg and 20 mg/kg body weight of mice) based on a previous study [28], for four days prior to the administration of 2.5% dextran sodium sulfate solution, and continued for another seven days along with DSS (Figure 1A). The severity of the DSS-induced colitis, disease activity index (DAI), colon length, macroscopic score, and histological score was significantly improved by saffron at a 20 mg/Kg dose while saffron at the dose of 10 mg/Kg showed improvement in the DAI, macroscopic score, and histological score (Figure 1B–F). The results indicate that saffron reduces the severity of DSS-induced colitis in mice by improving the gross and histopathological characteristics of the colonic mucosa.

### 2.2. Saffron Prevented Increase in DSS-Induced Pro-Inflammatory Cytokines and 5-HT Level in Mice Colonic Tissue

**Figure 2:**
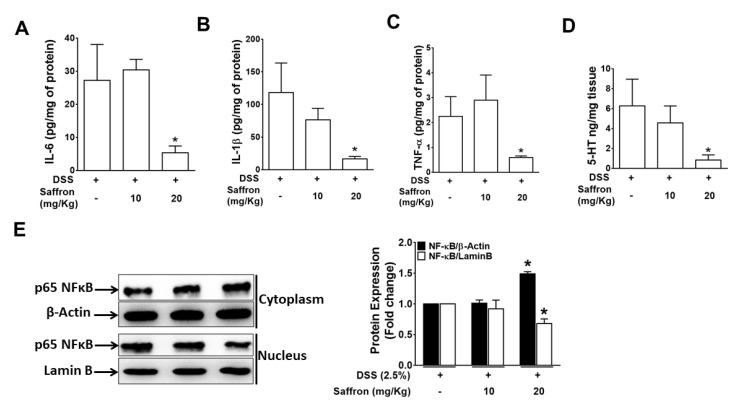
Pre-treatment with saffron inhibited the DSS-induced secretion of pro-inflammatory cytokines and the serotonin level in colon tissue. The supernatants from the homogenized colon tissue were analyzed for (**A**) IL-6, (**B**) IL-1β, (**C**) TNF-α, and (**D**) Serotonin (5-HT). Data represent the mean ± SEM (*n* = 4/group). * *p* < 0.05, compared to the vehicle-treated mice. (**E**) Cytoplasmic and nuclear protein extracted from colon tissues were analyzed for NF-κB. Representative blots of the cytoplasmic and nuclear NF-κB from the colon tissue of three random mice from each group. The bar graph represents the mean ± SEM. * *p* < 0.05, compared to the vehicle-treated mice.

A number of different immune cells, such as macrophages and dendritic cells, promote the recruitment of additional immune cells to inflamed tissue. [29,30]. The increase in the pro-inflammatory cytokines such as IL-6, IL-1β, and TNF-α in tissue indicates an aggravated immune response at the site of inflammation. Therefore, we analyzed the secreted pro-inflammatory cytokines in colonic tissue of mice and found that the DSS-induced increased secretion of IL-6, IL-1β, and TNF-α was significantly reduced by saffron (20 mg/kg), reducing the severity of the DSS-induced colitis. (Figure 2A–C). Additionally, we found that the DSS-induced increased 5-HT level in the colon tissue was significantly inhibited by the saffron at a higher dose (Figure 2E), further supporting the beneficial effect of saffron in preventing colitis. To investigate the mechanism by which saffron suppressed the DSS-induced pro-inflammatory cytokines, we analyzed NF-κB in the cytoplasmic and nuclear protein of the colon tissues. The nuclear translocation of NF-κB by DSS was significantly decreased by a higher dose of saffron in mice (Figure 2E). These data indicate that saffron inhibits DSS-induced secretion of pro-inflammatory cytokines in mice colons by decreasing the nuclear translocation of NF-κB.

### 2.3. Saffron Alters the Mouse Gut Microbiota Composition in DSS-Treated Mice

**Figure 3:**
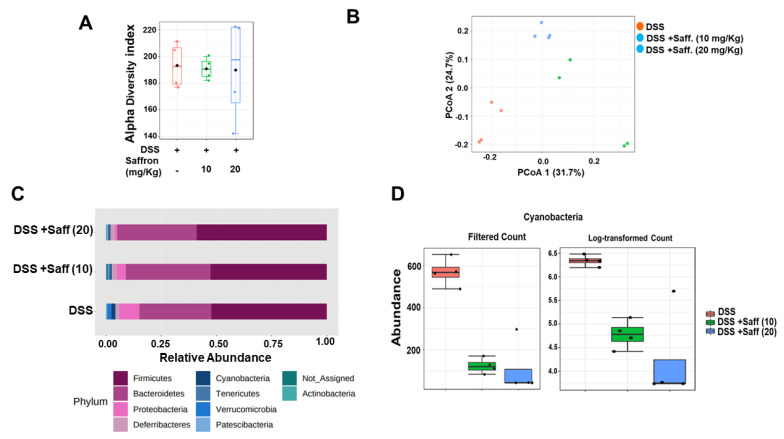
Microbial composition analysis in the cecal samples of saffron pre-treated and untreated mice challenged with DSS. The cecal content of mice were subjected to 16S rRNA partial sequencing profiling analysis and the figure represents (**A**) the alpha diversity of the three group of mice using the Chao1 index. (**B**) PCoA of the Bray–Curtis dissimilarity, showing distinct microbiota in each group of mice. (**C**) Taxonomic summaries at the phylum level. (**D**) Abundance of Cyanobacteria in the saffron-treated and untreated groups (*n* = 4/group).

To determine whether saffron alters the mouse gut microbiota composition, we analyzed the cecal microbial composition in three different groups of mice which received DSS, DSS plus 10 mg/kg of saffron, and DSS plus 20 mg/kg of saffron. As shown in Figure 3A, the alpha diversity of the three groups was not different. However, the three groups of mice were separated into distinct clusters, as shown by visualization of the Bray–Curtis diversity by principal coordinate analysis (PCoA) (Figure 3B; *p* < 0.05). The two groups of saffron-treated mice appeared more similar in composition while largely differed when compared to the DSS-treated group. In addition, the taxonomic summaries (average of each group) at the phylum level revealed greater similarity between the saffron-treated mice. Moreover, saffron administration depleted the Proteobacteria phylum, in which the effect seems to be much greater at 20 mg/kg compared to 10 mg/kg of saffron (Figure 3C; *p* < 0.05); in turn, this phylum is absent in naïve SPF mice [31]. Similarly, we observed that, at the operational taxonomic unit (OTU) level, Cyanobacteria were depleted in the saffron-treated mice and the effect was greater at the dose of 20 mg/kg (Figure 3D, *p* < 0.05).

### 2.4. Saffron Increased Beneficial Short-Chain Fatty Acids (SCFAs) in DSS-Treated Mice

**Figure 4:**
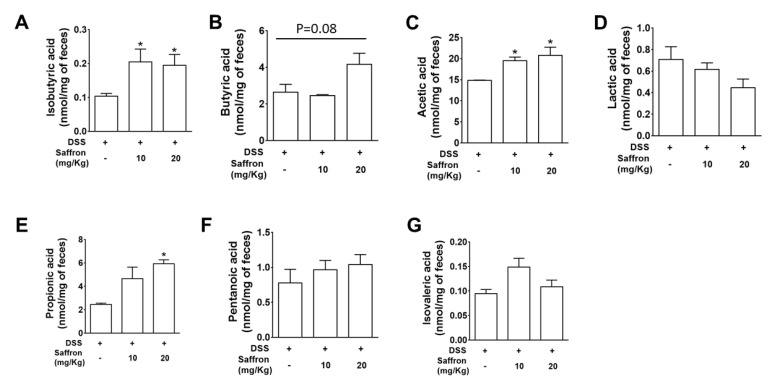
Saffron prevented depletion of the SCFA concentrations in fecal samples of DSS-treated mice. Fecal samples of mice were analyzed for determining the concentration of (**A**) isobutyric acid, (**B**) butyric acid, (**C**) acetic acid, (**D**) lactic acid, (**E**) propionic acid, (**F**) pentanoic acid, and (**G**) isovaleric acid. Data represent the mean ± SEM (*n* = 4/group). * *p* < 0.05, compared to the vehicle-treated mice fecal sample.

SCFAs are vital for regulation of intestinal epithelial cell (IEC) functioning, to modulate their proliferation, differentiation, and promoting gut barrier function. SCFAs are known to be altered by a change in the microbiota composition [32]. To confirm the functional effect of the altered microbiota by saffron administration during DSS-induced colitis, we analyzed the SCFA levels in the feces by using gas chromatography–mass spectrometry (GC/MS). We found that saffron at both doses significantly increased isobutyric acid and acetic acid, while at higher dose it also increased propionic acid in feces (Figure 4).

## 3. Discussion

The application of natural and traditionally trusted medicinal product provides a safe alternative to manage inflammatory conditions in the gut. Saffron has been used since ancient days in diets, and its various components, such as crocin, crocetin, picrocrocin, and safranal, are reported to have significant efficacy in peptic ulcer, stomach cancer, ulcerative colitis, colorectal cancer, and pancreatic disorder [15,33,34]. A randomized, double blind, placebo control trial conducted on mild to moderate ulcerative colitis patient suggested that dietary saffron may be effective in reducing the severity of disease in UC patients by improving the antioxidant factors [33]. Some other studies illustrated the protective effects of the active constituents of saffron, such as crocin and safranal, against experimental colitis [35,36]. In the present study, we investigated the effect of unfractionated saffron in preventing DSS-induced colitis in mice by evaluating its effect on the serotonin level, microbiota, and short-chain fatty acids. We found that supplementation of saffron along with diet reduced the severe effect of DSS. A previous study reported slight, although not significant restoration of colon length and percentage of weight loss in the safranal (200 mg/kg, 500 mg/kg)-treated groups of mice, with a significant decrease in the DAI score [37]. We found that the unfractionated saffron (20 mg/kg) treatment significantly increased the colon length, decreased the DAI, and improved the histopathological characteristics of the colonic mucosa, exhibiting its protective effects during experimental colitis. The tissue levels of pro-inflammatory cytokines, such as IL-6, IL-1β, and TNF-α, were significantly reduced in mice treated with saffron at the dose of 20 mg/Kg (Figure 2A–C). This effect of saffron may be achieved by the virtue of its antioxidant property. NF-κB, being a redox-sensitive transcription factor, is activated by various inflammatory insults and translocate to the nucleus to induce proinflammatory cytokines during inflammation [16,17]. Saffron pre-treatment inhibited the nuclear translocation of p65 NF-κB in mice colonic tissue samples, which corresponds to inhibition of the secretion of IL-6, IL-1β, and TNF-α. Saffron at the same dose significantly inhibited the serotonin level in colon tissue (Figure 2D). Serotonin was previously demonstrated to play a key role in the pathogenesis of experimental colitis by priming colon epithelial cells to inflammation. It has been previously revealed that serotonin modulates the gut function and gut microbiota composition by selecting colitogenic microbiota [38]. The amount of serotonin was significantly decreased in the saffron-pretreated mice, and it can be speculated that saffron may inhibit the oxidative stress probably by activating an antioxidant mechanism [33]. Additionally, disturbance in gut microbiota composition is largely associated with various diseases, as gut microbiota perform various important functions, such as digestion of polysaccharides, vitamin synthesis, and boosting of the immune system [39,40]. There were no significant changes in the alpha diversity of the gut microbiome, but each group showed distinct clusters while analyzing beta diversity (Figure 3B). The group that only received DSS was different compared to the mice that received saffron. An increased abundance of Proteobacteria has been implicated in a Crohn’s disease and its load has been suggested as a potential criterion in the diagnosis of dysbiosis in gut microbiota [41]. Saffron-treated mice showed depletion in the Proteobacteria phylum. Furthermore, unlike in human IBD, Cyanobacteria, which are reported to increase in DSS-induced colitis [42,43], which is in agreement with our study, were depleted by saffron as observed at the OTU-level analysis (Figure 3C,D). This reduction in colitogenic bacteria (Proteobacteria and Cyanobacteria) is likely to be sensed directly by the immune system, leading to a reduction in pro-inflammatory signals and markers. However, further in-depth studies are warranted to validate these findings. SCFAs, such as butyric acid, acetic acid, and propionic acid, are reported to participate in controlling inflammation and repair the colon epithelium [32]. SCFA levels are known to be reduced in fecal samples of IBD patients and in experimental colitis [44,45,46]. Saffron preserved the essential SCFAs in mice feces, indicating its beneficial attributes in maintaining colonic microbial populations during colitis. The cumulative effects of saffron treatment, leading to the positive changes in observed macroscopic, histological, and immune markers, have potential translational implications for patients with intestinal inflammation. In fact, based on our previous and the present findings [28], we calculated the human equivalent dose (20 mg/Kg) of saffron that showed the best outcome in DSS-induced colitis mice and started a clinical trial in patients with mild to moderate ulcerative colitis. We have already registered our clinical trial for this application at clinicaltrials.gov (https://www.clinicaltrials.gov/ct2/results?cond=saffron&term=&cntry=&state=&city=&dist= accessed on 1 June 2021) (NCT04749576).

## 4. Materials and Methods

### 4.1. Mice

Age-matched C57BL/6N mice were purchased from Taconic Biosciences (Rensselaer, NY, USA). All experimental animal procedures were in accordance with the guidelines and principles of the Canadian Council of Animal Care and were approved by the Animal Care Committee McMaster University (AUP # 19-02-09).

### 4.2. Pre-Treatment with Saffron and Evaluation of DSS-Induced Colitis

Saffron aqueous extract at two different doses (10 mg and 20 mg per kg body weight) were given to the mice (N = 4 mice/group) by oral gavage for four days prior to the administration of DSS (mol wt. 36–54 kilo daltons; ICN Biomedicals Inc., Soho, OH, USA) in their drinking water at 2.5% weight/volume (*w*/*v*) along with saffron for 7 more days. The average DSS consumption per cage was recorded every day for the duration of the experiment. Mice were sacrificed on the 7th day after the beginning of DSS administration to examine the severity of colitis using previously published scoring systems. The disease activity index (DAI) was calculated using the scores of body weight loss, bloody feces, and consistency of stool. Macroscopic scoring was done after the mice were sacrificed by careful observation of rectal bleeding, rectal prolapse, colonic bleeding, and diarrhea. Colonic histological damage score was based on goblet cell depletion, the loss of crypt architectures, inflammatory cell infiltration, and crypt abscess.

### 4.3. Enzyme Linked Immunosorbent Assay (ELISA)

Colon tissues from mice in each group were homogenized in tissue lysis buffer and the supernatant were used to analyze the level of IL-1β, IL-6, and TNF-α using commercially available ELISA kits from R&D System Inc. (Minneapolis, MN, USA) and expressed in units/mg of protein.

The serotonin level in tissue were measured as previously described [26], using commercially available enzyme-linked immunosorbent assay (ELISA) kits (Cat. # IM1749; Beckman Coulter, Fullerton, CA, USA). The serotonin level was expressed as a function of tissue weight (ng/mg).

### 4.4. Western Blot

Cytoplasmic and nuclear proteins were extracted by using the NE-PER nuclear and cytoplasmic extraction reagent kit (no. 78833, Thermo Scientific, Waltham, MA, USA) as described earlier [5]. Briefly, the protein concentration in the extract was determined by the DC Protein Assay Kit (Bio-Rad Laboratories, Mississauga, ON, Canada). Protein samples were separated using sodium dodecyl sulfate-polyacrylamide gel electrophoresis and were electrophoretically transferred onto nitrocellulose or polyvinylidene difluoride membranes. The membranes were incubated with 5% bovine serum albumin (BSA) in s 1× Tris-buffered saline Tween 20 at room temperature for 1 h and then probed with primary antibodies overnight at 4 °C. The membranes were then washed 3 times with Tris-buffered saline containing 0.1% Tween 20 followed by incubation with corresponding secondary antibodies for 1 h at room temperature. Immunodetection was performed by visualization of the membrane using a chemiluminescent reagent (Thermo Scientific) and by exposure to a luminescent image analyzer, the ChemiDoc Touch Imaging System (Bio-Rad Laboratories, Hercules, CA, USA). NF-κB p65 (1:1000; catalog no. ab16502) and lamin B1 (1:1000; catalog no. ab65986) were purchased from Abcam (Cambridge, MA, USA). β-actin (1:1000; catalog no. 4970) was purchased from Cell Signaling Technology, Inc. (Boston, MA, USA). The rabbit polyclonal antibody was obtained from Abbiotec (San Diego, CA, USA).

### 4.5. Bacterial Diversity and Profiling Analysis of the Cecal Microbiota

Bacterial profiling of cecal samples was carried out by amplification of the V3–V4 regions of the 16S rRNA gene, as described previously [47,48]. Amplification products were sequenced on an Illumina MiSeq with 2 × 250 nt paired end reads. The OTU abundance table obtained were given as input to Microbiome Analyst using default parameters and rarefying the data to the minimum library size with total sum normalization. The low variance filter was set at 10% using the inter-quartile range, and 20% prevalence was kept with four minimum counts [49]. The Microbiome Analyst platform was used to analyze alpha and beta diversities, and to compare the relative abundance of taxa at the phylum level [49].

### 4.6. Analysis of Fecal Short-Chain Fatty Acid Using Gas Chromatography—Mass Spectrometry

The concentrations of SCFAs in feces of mice were determined by gas chromatography–mass spectrometry, as described previously [26]. Briefly, e-tubes in which fecal samples were acidified with a weight equivalent amount of 3.7% hydrochloric acid were sonicated in methanol for 20 min before use. To the acidified samples, internal standards (14.72 mmol/L butyric acid-d_7_) were added, followed by the addition of diethyl ether to obtain a diethyl ether–fecal extract. The acidified samples were extracted three times with propyl formate containing butyric acid-d_7_ as the internal standard, and a 60 µL extract aliquot was derivatized with 25 µL MTBSTFA at 40 °C for 1 h and then analyzed by GCMS Then the derivatized samples were run through the 6890N Network GC system (Agilent Technologies, Mississauga, ON, Canada) equipped with DB-17HT (30 m × 0.25 mm ID, 0.15 mm film) and 5973N Mass Selective Detector (Agilent Technologies). Acetic acid, propionic acid, isobutyric acid, butyric acid, isovaleric acid, pentanoic acid, and lactic acid were quantified and reported as nmol/mg of fecal sample. The calibration curves were obtained for all seven targets by injecting all the standards as a mixture.

### 4.7. Statistical Analysis

Student’s *t*-test or one-way ANOVA in GraphPad Prism ver. 9.0 (San Diego, CA, USA) was used to determine the significance of the intergroup differences. Data are expressed as the mean ± SEM. *p* values of less than 0.05 were considered statistically significant.

## 5. Conclusions

Our data suggest that saffron exhibits its prophylactic effect on DSS-induced colitis in mice by reducing the serotonin levels, inhibiting pro-inflammatory cytokine secretion, and maintaining the diversity of the gut microbiota and the SCFA level. Our pre-clinical study provides an alternative and safe approach to reduce the susceptibility to GI disorders, including IBD, by incorporating saffron, a natural and edible product, into diets. However, a well-designed clinical trial may shed some light on the efficacy of saffron in different GI disorders, such as IBD.

## Data Availability

The data presented in this study are available on request from the corresponding author.

## Sample Availability

Samples are available from the authors.
